# Altered expression of miRNAs and methylation of their promoters are correlated in neuroblastoma

**DOI:** 10.18632/oncotarget.13090

**Published:** 2016-11-04

**Authors:** Marco Maugeri, Davide Barbagallo, Cristina Barbagallo, Barbara Banelli, Stefania Di Mauro, Francesco Purrello, Gaetano Magro, Marco Ragusa, Cinzia Di Pietro, Massimo Romani, Michele Purrello

**Affiliations:** ^1^ Dipartimento di Scienze Biomediche e Biotecnologiche, Sezione di Biologia e Genetica G Sichel, Unità di BioMedicina Molecolare, Genomica e dei Sistemi Complessi, Università di Catania, Catania, Italy, EU; ^2^ UOS Epigenetica dei Tumori, IRCCS A.O.U. San Martino-IST, Genova, Italy, EU; ^3^ Dipartimento di Biomedicina Clinica e Molecolare, Università di Catania, Ospedale Garibaldi, Catania, Italy, EU; ^4^ Dipartimento di Scienze Mediche, Chirurgiche e Tecnologie Avanzate G.F. Ingrassia, Università di Catania, Catania, Italy, EU; ^5^ Department of HealthSciences, University of Genova, Genova, Italy, EU

**Keywords:** miRNAs encoding genes, promoter methylation profiles, neuroblastoma, gene expression, 5'-AZA

## Abstract

Neuroblastoma is the most common human extracranial solid tumor during infancy. Involvement of several miRNAs in its pathogenesis has been ascertained. Interestingly, most of their encoding genes reside in hypermethylated genomic regions: thus, their tumor suppressor function is normally disallowed in these tumors. To date, the therapeutic role of the demethylating agent 5′-Aza-2 deoxycytidine (5'-AZA) and its effects on miRNAome modulation in neuroblastoma have not been satisfactorily explored. Starting from a high-throughput expression profiling of 754 miRNAs and based on a proper selection, we focused on miR-29a-3p, miR-34b-3p, miR-181c-5p and miR-517a-3p as candidate miRNAs for our analysis. They resulted downregulated in four neuroblastoma cell lines with respect to normal adrenal gland. MiRNAs 29a-3p and 34b-3p also resulted downregulated *in vivo* in a murine neuroblastoma progression model. Unlike the amount of methylation of their encoding gene promoters, all these miRNAs were significantly overexpressed following treatment with 5′-AZA. Transfection with candidate miRNAs mimics significantly decreased neuroblastoma cells proliferation rate. A lower expression of miR-181c was significantly associated to a worse overall survival in a public dataset of 498 neuroblastoma samples (http://r2.amc.nl). Our data strongly suggest that CDK6, DNMT3A, DNMT3B are targets of miR-29a-3p, while CCNE2 and E2F3 are targets of miR-34b-3p. Based on all these data, we propose that miR-29a-3p, miR-34b-3p, miR-181c-5p and miR-517a-3p are disallowed tumor suppressor genes in neuroblastoma and suggest them as new therapeutic targets in neuroblastoma.

## INTRODUCTION

Neuroblastoma is a neuroectodermal tumor that originates from precursor cells of the sympathetic nervous system. It represents the third leading cause of cancer-related deaths in childhood [1]. Its heterogeneous clinical phenotype, ranging from rapid progression to spontaneous regression, is due to the biological and genetic features of the tumor. The prognosis of stage I-III neuroblastoma (with a tumor confined to the originating organ or its surrounding tissues) is quite favorable, whereas that of metastatic stage IV is dismal. Stage IV-S neuroblastoma is a metastatic disease seen exclusively in infants: its metastases spread out very rapidly, but surprisingly it regresses spontaneously with a high survival rate [2]. The most frequently characterized cytogenetic alterations in neuroblastoma include: (i) amplification of the gene encoding MYCN (a protooncogenic transcription factor), localized at 2p24; (ii) loss of heterozygosity (LOH) or rearrangements of the distal portion of 1p31-ter, 3p22, 11q23; (iii) gain of chromosome arms 1q or 17q. Besides these abnormalities, gain of chromosomes 4q, 6p, 7q, 11q and 18q, amplification of MDM2 and MYC genes, and LOH at 14q, 10q, 19q13 have also been described [3, 4]. More recently, the involvement of miRNAs in neuroblastoma pathogenesis has been assessed [5, 6]. Deregulation of miRNAs expression in malignant neuroblastomas may be due to several factors, as MYCN amplification, chromosomal deletions, or abnormal epigenetic regulation [7]. Promoters of tumor suppressor miRNAs are commonly hypermethylated in cancer [8–12]. It is known that reversion of this hypermethylation status could lead to reactivation of their tumor suppressor function in cancer cells [13–15]. The well-known demethylating agent 5′-Aza-2 deoxycytidine (5'-AZA) and its deoxy derivative (Decitabine) are currently used in the treatment of myelodysplastic syndrome (FDA approved) [16] and have been suggested for therapy of diffuse large B-cell lymphoma [10]. Apart from a published study [17], to date neither the therapeutic role of 5′-AZA nor its effects on miRNAome modulation have been appropriately investigated in neuroblastoma. To clarify the role of miRNAs in neuroblastoma, we sought to: (1) investigate the effects of 5′-AZA on the global expression of miRNAs in five commonly used neuroblastoma cell line models; (2) analyze the relationship between the expression of four prioritized miRNAs and the methylation status of their encoding gene promoters; (3) identify and characterize the expression of the most interesting among their targets; (4) assay their involvement in neuroblastoma cell viability; (5) analyze the prognostic value of altered expression of miRNAs and their targets.

## RESULTS

### MiRNAome expression in neuroblastoma cells after treatment with 5′-AZA

Treatment with 5′-AZA caused remarkable alterations of miRNAome expression in all neuroblastoma cell lines: more than 60% differentially expressed (DE) miRNAs were upregulated (Supplementary Table S1). We focused our analysis on 12 miRNAs (miR-22, miR-29a-3p, miR-34a, miR-126, miR-140-3p, miR-141, miR-181c-5p, miR-202, miR-455-5p, miR-508-3p, miR-517a-3p and miR-576-3p). This choice was based on: (i) their differential expression; (ii) their inclusion in hypermethylated CpG islands in neuroblastoma or other neoplasias; (iii) known overexpression after treatment with demethylating agents in various types of cancers; (iv) known downregulation in neuroblastoma; (v) functional relationship with MYCN. These miRNAs were identified as DE and upregulated in at least three neuroblastoma cell lines (Supplementary Table S2). The promoter of the genes encoding seven of them (miR-29a-3p, miR-34a, miR-126, miR-141, miR-181c-5p, miR-202 and miR-517a-3p) contained CpG islands, whose methylation significantly decreased after treatment with 5′-AZA (see later). Apart from intronic miR-126, all other miRNAs are intergenic. A previous report by Watanabe K et al. demonstrates that miR-126 expression may be regulated by the methylation status of an upstream CpG island, located within an intron of its host gene EGFL7 [18]. Single TaqMan expression assays (STAs), extended to miR-34b (another member of the miR-34 cluster), revealed that miR-29a-3p, 34b-3p, 181-c-5p and 517a-3p are upregulated in at least three different neuroblastoma cell lines (Table 1).

**Table 1:** DE miRNAs after treatment with 5′-AZA

DE miRNA	Cell line	RQ vs miR-24	RQ vs U6
miR-29a-3p	ACN	1.91	1.82
	GIMEN	1.34	2.45
	SH-SY5Y	1.7	1.8
	SK-N-BE(2)-C	2.3	2.25
	SK-N-SH	2.91	2.78
miR-34a	ACN	2.65	2.52
	GIMEN	2.17	3.98
	SH-SY5Y	0.32	0.88
	SK-N-BE(2)-C	0.55	0.54
	SK-N-SH	0.56	0.54
miR-34b-3p	ACN	5.21	4.97
	GIMEN	1.43	2.61
	SH-SY5Y	0.17	0.47
	SK-N-BE(2)-C	5.68	5.56
	SK-N-SH	1.75	0.72
miR-126	ACN	12.47	11.9
	GIMEN	3.23	5.91
	SH-SY5Y	0.97	2.67
	SK-N-BE(2)-C	0.18	0.18
	SK-N-SH	1.17	1.12
miR-141	ACN	2.24	2.13
	GIMEN	3.51	6.44
	SH-SY5Y	0.15	0.41
	SK-N-BE(2)-C	0.87	0.85
	SK-N-SH	1.07	1.03
miR-181c-5p	ACN	2.11	2.01
	GIMEN	2.08	3.81
	SH-SY5Y	1.79	4.9
	SK-N-BE(2)-C	1.64	1.61
	SK-N-SH	1.19	1.14
miR-202	ACN	1.46	1.39
	GIMEN	4.82	8.84
	SH-SY5Y	0.59	1.61
	SK-N-BE(2)-C	1.46	1.43
	SK-N-SH	1.3	1.25
miR-517a-3p	ACN	1.06	1.01
	GIMEN	1.54	1.5
	SH-SY5Y	1.99	2.71
	SK-N-BE(2)-C	1.77	1.73
	SK-N-SH	2.06	1.97

### CpG island methylation status of DE miRNAs encoding genes following 5′-AZA treatment

Treatment with 5′-AZA determined a statistically significant decrease of the methylation of specific CpG islands, located upstream to the promoter of the genes encoding miR-29a, miR-34b/c, miR-126, miR-181c/d, miR-200c/141, miR-202 and miR-517a (Table 2). Detailed data on the methylation status within the CpG islands analyzed, before and after 5′-AZA treatment, are reported in Supplementary Table S3.

**Table 2:** Percentage of methylation of CpG islands, before (control) and after 5′-AZA treatment

	miR-29a		miR-34b/c		miR-126		miR-181c/d		miR-200c/141		miR-202		miR-517a	
NB cell lines	Control (%)	5'Aza (%)	Control (%)	5'Aza (%)	Control (%)	5'Aza (%)	Control (%)	5'Aza (%)	Control (%)	5'Aza (%)	Control (%)	5'Aza (%)	Control (%)	5'Aza (%)
**ACN**	85	80	44	38	46	43	59	47	84	70	34	35	80	78
**GI-ME-N**	90	73	88	79	84	66	94	62	86	65	88	66	96	77
**SK-N-BE(2)-C**	89	73	93	76	48	39	88	60	89	60	88	73	90	80
**SK-N-SH**	85	60	56	44	34	25	87	52	86	54	85	53	88	66
**SH-SY5Y**	84	58	13	10	86	53	92	49	96	54	74	60	95	72
**p-value**	0.009		0.02		0.05		0.004		0.004		0.04		0.02	

### Expression of candidate miRNAs in murine neuroblastoma biopsies

Sequences of miR-29a-3p, 34b-3p and 181c-5p are perfectly conserved between humans and rodents. The data reported by Beckers et al., following R2 microarray and visualization platform analyses (see Materials and Methods), showed a marked downregulation of miRNAs 29a-3p and 34b-3p in mouse neuroblastoma samples with respect to controls in a neuroblastoma progression model (Supplementary Figure S1) [19]. The same dataset did not show any significant variation in the expression of miR-181c-5p. Data on the expression of miR-517a-3p are not available due to the absence of the gene encoding this miRNA in the mouse genome.

### Expression of candidate miRNAs in neuroblastoma cell lines

Expression profiling of candidate miRNAs in GI-ME-N, SK-N-BE(2)-C, SK-N-SH and SH-SY5Y revealed a statistically significant downregulation of miR-181c-5p and 517a-3p in all cell lines. MiR-29a-3p was downregulated in SK-N-BE(2)-C, SK-N-SH and SH-SY5Y. MiR-34b-3p was significantly downregulated in SK-N-BE(2)-C and GI-ME-N (Supplementary Figure S2A). In all these experiments, adrenal gland was used as calibrator tissue.

### Selection of DE miRNAs targets

**Figure 1:**
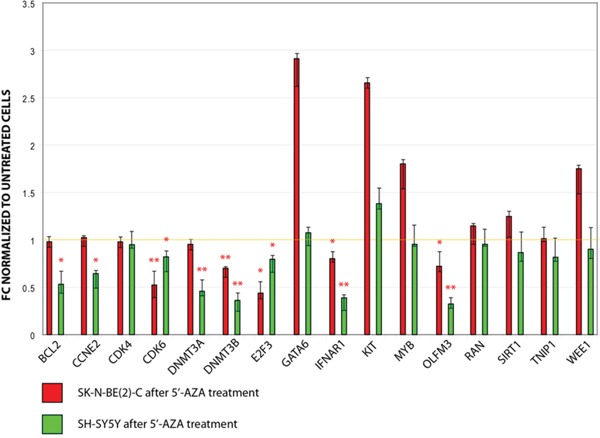
Expression of candidate miRNAs targets in SH-SY5Y and SK-N-BE(2)-C after treatment with 5′-AZA Values are reported as fold change (FC) versus untreated cells (controls). * p-value < 0.05; ** p-value < 0.01 (Student's t-test, n = 3).

*In silico* analysis of DE miRNAs targets allowed to select four validated targets for both miR-29a-3p (CDK6, DNMT3A, DNMT3B, RAN) and miR-181c-5p (BCL2, GATA6, KIT, SIRT); five validated targets for miR-34b-3p (BCL2, CCNE2, CDK4, E2F3, MYB); four predicted targets for miR-517a-3p (IFNAR1, OLFM3, TNIP1, WEE1) (Supplementary Table S4). Expression of these 16 targets was assayed in SH-SY5Y and SK-N-BE(2)-C after treatment with 5′-AZA. Eight targets resulted significantly downregulated after treatment with 5′-AZA: CDK6 and DNMT3B (validated targets of miR-29a-3p), E2F3 (validated target of miR-34b-3p), and OLFM3 and IFNAR1 (predicted targets of miR-517a-3p) were downregulated in both cell lines. DNMT3A (validated target of miR-29a-3p), BCL2 (validated target of both miR-34b-3p and miR-181c-5p), CCNE2 (validated target of miR-34b-3p) were downregulated only in SH-SY5Y (Figure 1).

### Targets expression in SK-N-BE(2)-C and SH-SY5Y transfected with miRNAs mimics

**Figure 2:**
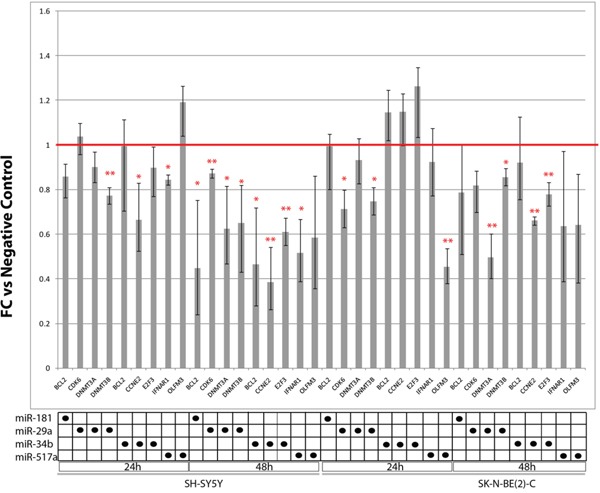
Expression of candidate miRNAs targets in SH-SY5Y and SK-N-BE(2)-C transfected with miRNAs mimics for 24 h and 48 h Values are reported as fold change (FC) versus scramble-transfected cells (negative controls). * p-value < 0.05; ** p-value < 0.01 (Student's t-test, n = 3).

Efficiency of SK-N-BE(2)-C and SH-SY5Y transfection with miRNAs mimics is shown in Supplementary Figure S3. Only replicates with a transfection efficiency > 80% were considered for downstream assays. CDK6, DNMT3A, DNMT3B (targets of miR-29a-3p) and CCNE2, E2F3 (targets of miR-34b-3p) were downregulated in both cell lines after transfection with the respective miRNAs mimics, compared to matched scramble-transfected cells in at least one time point (Figure 2). CDK6, DNMT3A and DNMT3B show conserved miR-29a-3p binding sites as retrieved through TargetScan (Supplementary Table S5). Alignments among miRNAs and their targets revealed by microRNA.org are shown in Supplementary Figure S4.

### Expression of candidate miRNAs targets in neuroblastoma cell lines

CCNE2, CDK6, DNMT3B and E2F3 resulted overexpressed in SK-N-BE(2)-C, SK-N-SH and SH-SY5Y cell lines with respect to adrenal gland; DNMT3A was underexpressed in GIMEN, SK-N-BE(2)-C, SK-N-SH and SH-SY5Y cell lines (Supplementary Figure S2B). A negative correlation (even though statistically not significant) among miR-29a-3p, DNMT3A (r = −0.48) and DNMT3B (r = −0.60), as well as among miR-34b-3p and its candidate targets CCNE2 (r = −0.14) and E2F3 (r = −0.19) was observed. Analysis of *Tumor Neuroblastoma* - *SEQC* - *498* - *RPM* - *seqcnb1* dataset showed a significant negative correlation between miR-29a and its candidate targets DNMT3A (r = −0.110, p-value=0.01) and CDK6 (r = −0.129, p-value=4.1e-03).

### MiR-29a-3p, miR-34b-3p, miR-181c-5p and miR-517a-3p regulate neuroblastoma cell viability

**Figure 3:**
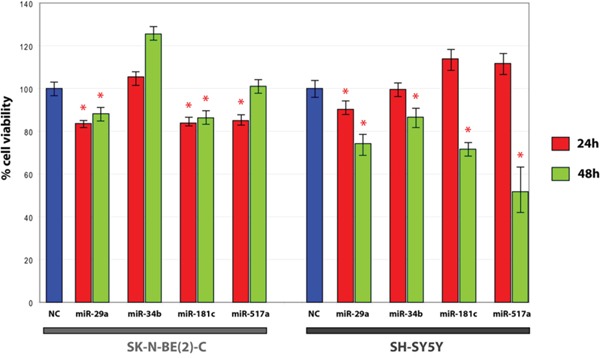
MTT assay in SH-SY5Y and SK-N-BE(2)-C transfected with candidate miRNAs mimics * p-value < 0.05 (Student's t-test, n = 6).

**Figure 4:**
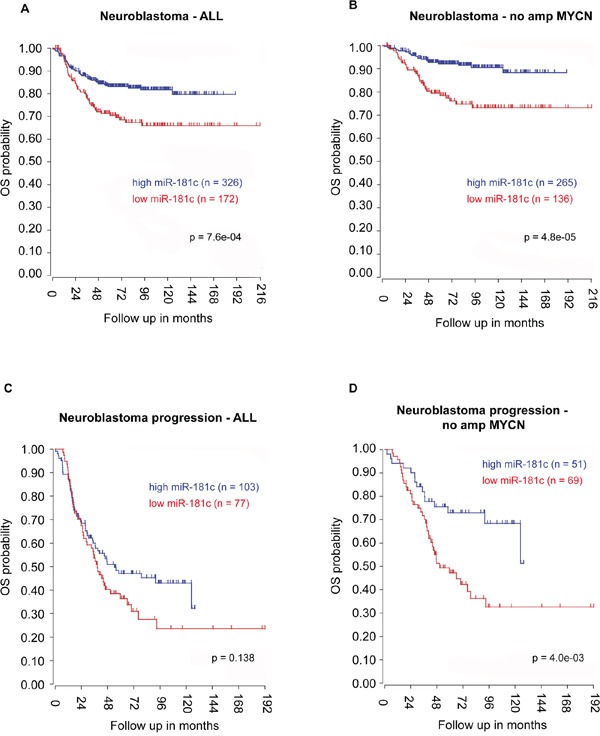
miR-181c expression and neuroblastoma patients' overall survival (OS) Lower expression of miR-181c is related with a worse OS, either **A.** in the whole cohort of neuroblastoma samples or **B.** in only no amplified MYCN cases. Reduced expression of miR-181c is not significantly associated to a worse prognosis when considering the whole cohort of patients who undergo tumor progression event **C.** this relationship is significant when considering only the cases that progress and have no amplification of MYCN **D.** The cut off modus for miR-181c expression to draw Kaplan-Meier curves derives from the scan setting.

Transfection with miR-29a-3p, miR-34b-3p, miR-181c-5p and miR-517a-3p mimics determined a significant decrease of cell viability, both in SK-N-BE(2)-C and in SH-SY5Y. The more pronounced decrease of cell viability was observed in SH-SY5Y, 48h after transfection with miR-517a-3p mimics (Figure 3). Interestingly, *Tumor Neuroblastoma* - *SEQC* - *498* - *RPM* - *seqcnb1* dataset analysis revealed that a decreased expression of miR-181c in neuroblastoma is linked to a worse overall survival (OS), either considering all neuroblastoma patients (χ^2^ = 11.34, df = 1, p-value = 7.6e-04, n = 498) or selecting only cases with no MYCN amplification (χ^2^ = 16.51, df = 1, p-value = 4.8e-05, n = 401) (Figure 4A, 4B). Moreover, by considering only neuroblastoma patients who showed relapse or progression of the disease and no MYCN amplification, lower expression of miR-181c was significantly associated with a worse prognosis (χ^2^ = 8.29, df = 1, p-value = 4.0e-03, n = 120) (Figure 4C). The latter association was not significant when considering the whole cohort of patients that undergoes progression or relapse of the disease (χ^2^ = 2.2, df = 1, p-value = 0.138, n = 180) (Figure 4D). The analysis performed for different neuroblastoma stages showed a significant association between decreased expression of miR-181c and a worse overall survival of stage 4 neuroblastoma cases with no MYCN amplification (χ^2^ = 7.17, df = 1, p-value = 7.4e-03, n = 116), but not with amplified MYCN (χ^2^ = 1.7, df = 1, p-value = 0.192, n = 65) (Supplementary Figure S5). Any significant association was lost when considering all the other neuroblastoma stages (1, 2, 3, 4s). By using the aforementioned dataset, we further observed a relationship between neuroblastoma progression and increased expression of CDK6 (χ^2^ = 27.50, df = 1, p-value = 1.6e-07), DNMT3A (χ^2^ = 14.56, df =1, p-value = 1.4e-04), DNMT3B (χ^2^ = 12.21, df = 1, p-value =4.8e-04), and E2F3 (χ^2^ = 161.81, df = 1, p-value =4.5e-37).

### Network analysis

**Figure 5:**
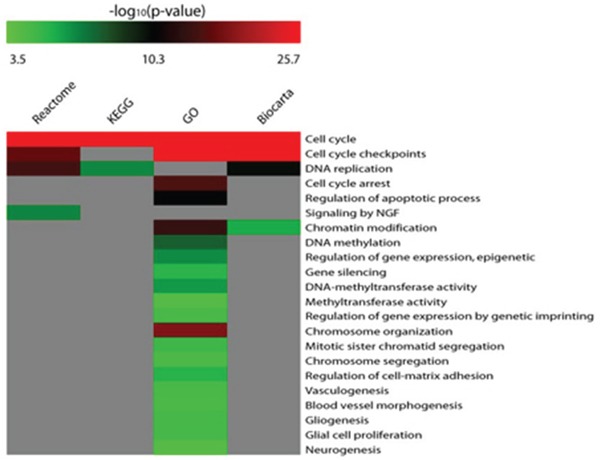
Matrix of enriched biological functions within the biological networks of DE miRNAs targets The higher is the p-value, the more significant is the enrichment in a specific pathway or Gene ontology (GO).

A network of protein-protein interactions was generated from nodes CDK6, DNMT3A, DNMT3B (targets of miR-29a-3p) and CCNE2 and E2F3 (targets of miR-34b-3p) and extended to their first neighbor interactants. This network comprises 120 nodes and 726 edges and it is centered on 21 hub nodes (See Materials and Methods). DNMT3A, DNMT3B and E2F3 are hubs of the network (Supplementary Table S6). Overall, the network is enriched in biological functions such as cell cycle, DNA methylation, neurogenesis (Hypergeometric test; Benjamini & Hochberg FDR Correction; p ≤ 0.01) (Figure 5).

## DISCUSSION

We investigated the involvement of miRNAs encoding genes and the role of their promoter methylation in neuroblastoma. Expression profiling of 754 miRNAs, combined with methylation assays of specific CpG islands and *in silico* analyses, allowed us to focus on miR-29a-3p, miR-34b-3p, miR-181c-5p and miR-517a-3p. MiR-29a-3p is known to be downregulated in neuroblastoma [20, 21] and several miR-29 family members are known to play a leading role in cancer etiology and pathogenesis [22]. According to public databases, our data strongly suggest CDK6, DNMT3A and DNMT3B as direct targets of miR-29a-3p: disallowed tumor-suppressive function of this miRNA may stimulate tumorigenesis by promoting cell cycle progression and altering *de novo* methylation of the genome. On the contrary, an experimental increase of miR-29a-3p levels is linked to decreased viability of neuroblastoma cells. Expression of miR-34b-3p is known to be epigenetically regulated by 5′-AZA [23], but to date its altered expression has not been associated with neuroblastoma. MiR-34a (another member of the miR-34 family) is a known tumor suppressor in neuroblastoma; it has been suggested as an epigenetic target for treatment of diffuse large B-cell lymphoma by 5′-AZA [10, 24, 25]. Our data suggest tumor-suppressor properties also for miR-34b-3p in neuroblastoma: its increased expression is linked to decreased levels of neuroblastoma cell viability. Based on our data and according to public databases, we suggest that CCNE2 and E2F3 are two candidate targets: interestingly, both genes are known to be involved in cell cycle progression. MiR-181c-5p is a known tumor suppressor in neuroblastoma: it belongs to the miR-181 family, whose members are known to be upregulated after MYCN silencing [26, 27]. Expression of miR-181c-5p and miR-517a-3p is known to be reactivated by 5′-AZA in gastric and bladder cancer, respectively [28, 29]. The link between miR-181c underexpression and poor outcome of neuroblastoma patients (found by querying public databases) suggests a potential prognostic value of this miRNA. Interestingly, this prognostic value seems to be restricted to stage 4 neuroblastoma patients and to be independent of MYCN amplification status, which alone is a known negative prognostic factor. A similar consideration may be made for CDK6, DNMT3A, DNMT3B and E2F3, which we propose as targets for miR-29a-3p and miR-34b-3p: their increased expression is related to poor prognosis. No links between miR-517a-3p and neuroblastoma have been published to date, notwithstanding it has been proposed as a tumor suppressor in several cancers [30, 31]. Our data convincingly suggest that this miRNA is a new tumor suppressor in neuroblastoma. Involvement of our candidate miRNAs in neuroblastoma pathways is also suggested by our *in silico* network analysis: nodes within the network were found to be enriched in several cancer-related biological functions, as well as in neurodevelopment. In conclusion, our experimental data demonstrate that miR-29a-3p, miR-34b-3p, miR-181c-5p and miR-517a-3p are involved in neuroblastoma and are potential new therapeutic targets in neuroblastoma.

## MATERIALS AND METHODS

### Cell cultures and treatments

Neuroblastoma cell lines ACN, GI-ME-N, SH-SY5Y, SK-N-BE(2)-C and SK-N-SH were provided by the Biologic Bank and Cell Factory of the IRCCS AOU San Martino-IST, Genova, Italy. Authenticity of cell lines was confirmed with Short Tandem Repeat (STR) analysis; cell lines were routinely tested and found free of mycoplasm contamination. ACN, GI-ME-N, SK-N-BE(2)-C and SK-N-SH were grown in RPMI 1640 medium (Sigma, S. Louis, MO, USA), whereas for SH-SY5Y cells DMEM medium (GIBCO) was used; tissue culture media were supplemented with 10% FBS (Sigma) and 2mM L-Glutamine (Lonza, Basel, Switzerland), 50 IU/mL sodium penicillin G, 50 μg/mL steptomycin sulphate (Cambrex, East Rutherford, NJ, USA), at 37°C under a water saturated 95% air- 5% CO2 atmosphere. SK-N-BE(2)-C, GI-ME-N, ACN and SH-SY5Y were treated in the presence or absence of 5μM 5′-AZA (Sigma) for 144 h, while SK-N-SH was treated for 72 h. Both medium and drug were refreshed daily.

### RNA isolation, reverse transcription and miRNAome profiling by TaqMan low density array

Total RNA was extracted by using TriZol (Lifetechnologies™, Carlsbad, CA, USA) and tested by Qubit (Lifetechnologies™) and spectrophotometry to assess its quantity and quality, respectively [32]. Total RNA from the adrenal gland was purchased from Takara Clontech^®^ (Mountain View, CA, USA): normal adrenal glands were pooled from 62 Caucasian males/females (15-61 years old), who had died of sudden death. We reverse transcribed 100 ng of total RNA with pools of miRNAs-specific primers; there were then pre-amplified [32]. Amplified products were loaded on TaqMan® Low Density Arrays (TaqMan® Human MicroRNA Array v3.0 A and B, Lifetechnologies™). To profile the expression of 754 miRNAs, Real-Time PCR was performed on a 7900HT Fast Real-Time PCR System (Lifetechnologies™).

### MiRNA expression data analysis

High-throughput real-time PCR data were quantitatively normalized by using the global median normalization method: Ct values from each sample were normalized to the median Ct of the array representing the sample. By performing Pearson's correlation between Ct values of each miRNA and median and mean Ct values of each array, we identified three RNAs (miR-24, RNU6 and RNU48), whose r-values were higher than 0.9. These RNAs were among the most stable within TLDAs by applying two different algorithms: DataAssist v.3 (Lifetechnologies™) and geNorm [33]. Accordingly, miR-24, RNU6 and RNU48 were selected as endogenous controls for downstream real-time PCR analysis. Relative quantities of miRNAs in treated and control samples were calculated by 2^−ΔΔCT^ method [34]. Statistically significant DE miRNAs were identified by SAM (Significance of Microarrays Analysis) algorithm (http://www.tm4.org), applying a two-class paired test on ΔCt values of treated and control samples. P-values were based on permutation (100 permutations), imputation engine used K-nearest neighbors (10 neighbors); false discovery rate (FDR) was tuned to < 0.15. We considered DE miRNAs those that: (1) were significantly DE by using all three endogenous controls; (2) had fold-changes (FC) <0.5 (underexpression) or >1.9 (overexpression); (3) whose raw data passed a quality check. Relative quantities (RQ) < 1 were converted to negative FC by the formula: FC = −1/RQ. Matrixes of miRNAome profiles were obtained by using ΔCt in MeV04 (http://www.tm4.org/mev.html) [32].

### Human and mouse datasets and survival analysis

By using R2 platform (http://r2.amc.nl), we analyzed the expression of miRNAs and their targets. We specifically used the following datasets: *Neuroblastoma Ghent* (Array Express: E-MTAB-2618) [19] and *Neuroblastoma SEQC* (GEO: GSE62564). Kaplan Mier curves were obtained through R2 as described by Matas-Rico E et al. [35].

### mRNA target expression analysis

MiRNA target expression was assayed by SYBR Green RT-PCR, according to the manufacturer's instructions (Lifetechnologies™). RQs and FCs of miRNA targets were calculated as reported above. HPRT was used as endogenous control. Primers to amplify miRNA targets were designed through Primer-BLAST (http://www.ncbi.nlm.nih.gov/tools/primer-blast/): their sequences are available upon request.

### DNA extraction and sodium bisulfite modification

Genomic DNA was extracted from 5′-AZA-treated and untreated (control) neuroblastoma cell lines through QIAamp DNA Mini Kit (Qiagen, Milan, Italy), according to the manufacturer's instructions. DNA was chemically modified with sodium bisulfite using the Epitect Bisulfite kit (Qiagen), according to the manufacturer's instructions [36].

### CpG island retrieval

EMBOSS Cpgplot (http://www.ebi.ac.uk/Tools/seqstats/emboss_cpgplot/) was used to retrieve CpG islands within a window of 10 Kbps upstream each miRNA genomic locus (considered criteria: Observed/Expected ratio > 0.60;Percent C + Percent G > 50.00; Length > 200).

### Pyrosequencing assay

PCR and sequencing primers were designed with the Pyrosequencing Assay Design Software (Qiagen) to recognize part of the CpG islands in the putative regulatory regions of the miRNAs analyzed. These primers were used to amplify and sequence the bisulfite-modified DNA from neuroblastoma cell lines. Pyrosequencing assays were performed with a SPQ 96MA instrument (Qiagen) and sequencing reactions were performed with the Pyro Gold reagent kit SPQ 96MA, according to manufacturer's instructions. Sequencing data were analyzed using the Pyro Q-CpG software (version 1.0.9).

### Selection of candidate miRNAs

Selection of candidate DE miRNAs, to be investigated through functional assays, was based on the following criteria: (i) RQ values (the more they are upregulated, the more they are considered as involved in the effectiveness of 5′-AZA treatment); (ii) their (or their validated or predicted targets) known involvement in cancer, as suggested by specialized databases (http://bioinfo.au.tsinghua.edu.cn/member/jgu/oncomirdb/) and literature; (iii) presence of CpG islands (validated or predicted, see later) within their promoter region; (iv) regulatory and functional interaction with the oncogene MYCN.

### Transfection with miRNA mimics

SK-N-BE(2)-C and SH-SY5Y cells were grown in 12- and 24-well plates, respectively, at a density of 1.2 x 10^5^ and 6 x 10^4^ cells per well. Cells were transiently reverse-transfected with 30 pmoles of miR-29a-3p, miR-34b-3p, miR-181c-5p and miR-517a-3p mimics or equal amounts of scrambled molecules for 24h and 48h, by using siPORTNeoFX Transfection Agent (Ambion^®^, Austin, TX), according to the manufacturer's instruction. All experiments were performed in biological triplicates.

### MTT assay

Cell viability was evaluated in miRNA mimics-transfected as well as in scramble-transfected cells through MTT assay, as previously described [37]. Briefly, 1.2 x 10^4^ cells / well were reverse-transfected with miR-29a-3p, miR-34b-3p, miR-181c-5p and miR-517a-3p mimics or equal amounts of scrambled molecules and were grown for 24h and 48h. Cell viability was evaluated at the end of each time point. All MTT experiments were performed in six biological replicates; statistical analysis was performed by unpaired T-test (<0.05).

### MiRNA target prediction

MiRecords (http://mirecords.biolead.org) and starBase (http://starbase.sysu.edu.cn) were initially queried to retrieve experimentally validated and predicted targets of DE miRNAs, by interpolating 11 different prediction tools. The list of predicted targets, common to miRecords and starBase, was further filtered based on negative correlation between their expression and that of miRNA: data of correlation were retrieved from MiRGator v3.0 (http://mirgator.kobic.re.kr). Evolutionarily conserved miRNA binding sites and miRNA/target alignments were retrieved using TargetScan release 7.1 (http://www.targetscan.org/vert_71/) and microrna.org (http://www.microrna.org/microrna/home.do), respectively.

### Functional enrichment of miRNAs' targets

Biological function of miRNA targets was investigated by the tools DAVID (https://david.ncifcrf.gov/home.jsp) and Fatigo (http://babelomics.bioinfo.cipf.es/): this approach led us to focus on targets specifically involved in cancer-related biological processes.

### Network generation and analysis

The biological network of protein-protein interactions was generated through MiMI plug-in [38] and visualized through Cytoscape v3.2.0 [39]. CDK6, DNMT3A, DNMT3B (targets of miR-29a-3p), CCNE2 and E2F3 (targets of miR-34b-3p) were given as input and interactions among them and their first interactants were automatically retrieved. Network centrality analysis was performed as previously described [37].

## SUPPLEMENTARY FIGURES AND TABLES










