# Supplementary material for: Altered expression of miRNAs and methylation of their promoters are correlated in neuroblastoma
Source: Oncotarget. 2016 Nov 4;7(50):83330–41. doi: 10.18632/oncotarget.13090 (PMC5347773; doi:10.18632/oncotarget.13090)
Supplement: Supplementary file 1 [file oncotarget-07-83330-s001.pdf]

Altered expression of miRNAs and methylation of their promoters are correlated in neuroblastoma

SUPPLEMENTARY FIGURES AND TABLES

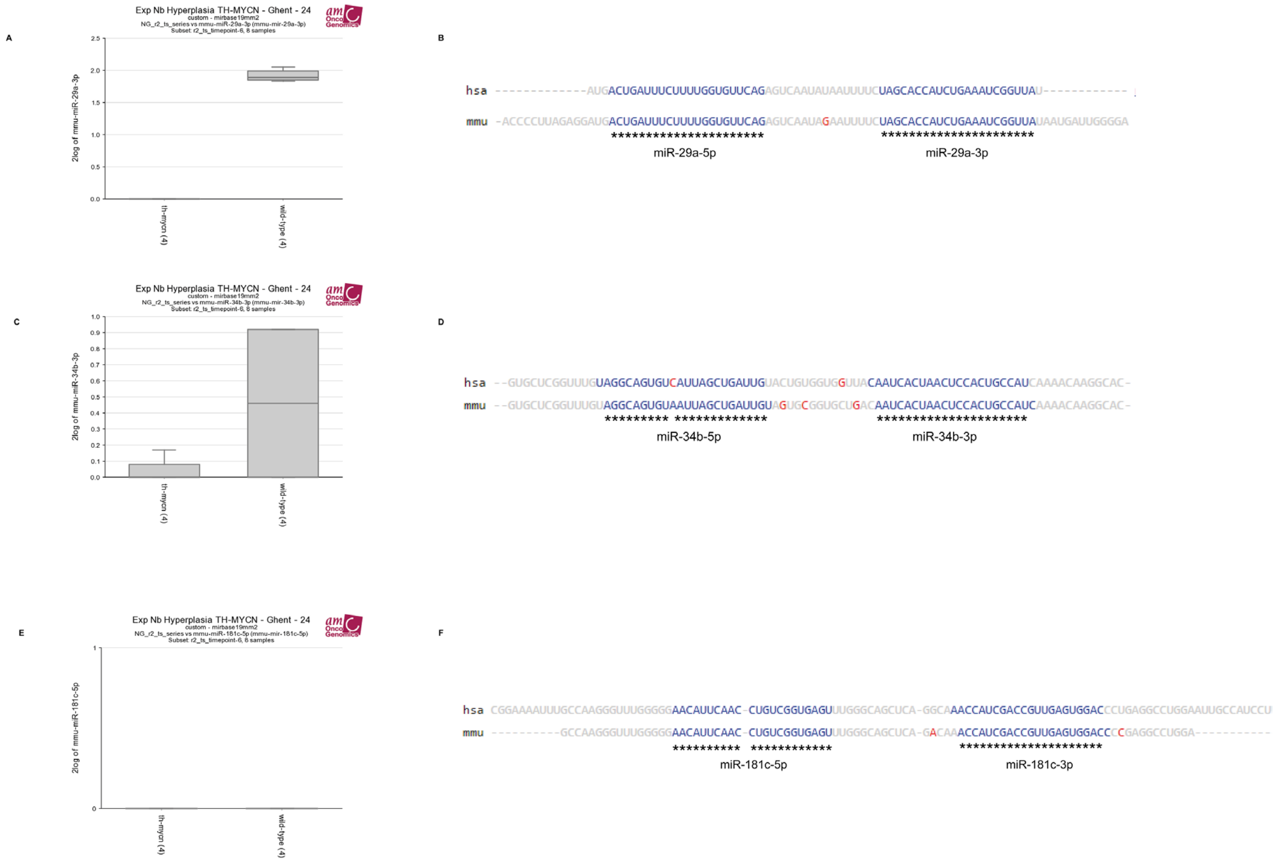

Supplementary Figure S1: Box plots of the expression of mmu-miR-29a-3p A. mmu-miR-34b-3p **C.** mmu-miR-181c-5p **E.** in neuroblastoma (th-mycn) and control (wild-type) samples (data from Neuroblastoma Ghent, Array Express: E-MTAB-2618, analyzed and visualized through R2 platform). Conservation of pre-miR-29a **B.** pre-miR-34b **D.** and pre-miR-181c **F.** sequences between *Homo sapiens* (hsa) and *Mus musculus* (mmu), identified through microRNAviewer (http://people.csail.mit.edu/akiezun/microRNAviewer/index.html).

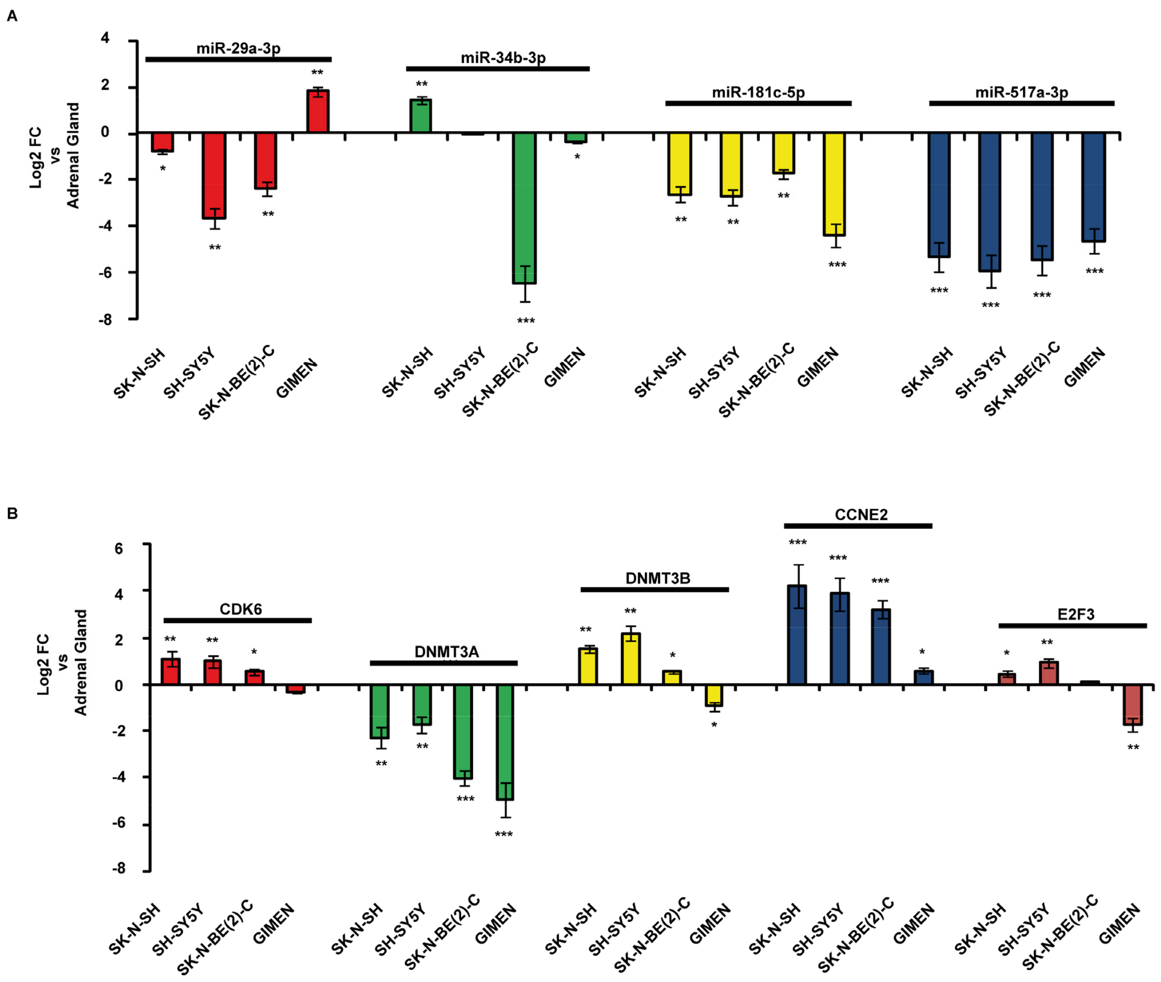

Supplementary Figure S2: Expression of miRNAs 29a-3p, 34b-3p, 181c-5p, 517a-3p A. and CDK6, DNMT3A/B (targets of miR-29a-3p), CCNE2, E2F3 (targets of miR-34b-3p) B. in GIMEN, SK-N-BE(2)-C, SK-N-SH, SH-SY5Y neuroblastoma cell lines. Expression values are reported as Log2 of the fold change (FC) versus the adrenal gland. Mir-191-5p and HPRT have been used as reference genes for miRNAs and protein-encoding genes assays, respectively. * p-value < 0.05, ** p-value < 0.01, *** p-value < 0.001, Student's t-test (n = 3).

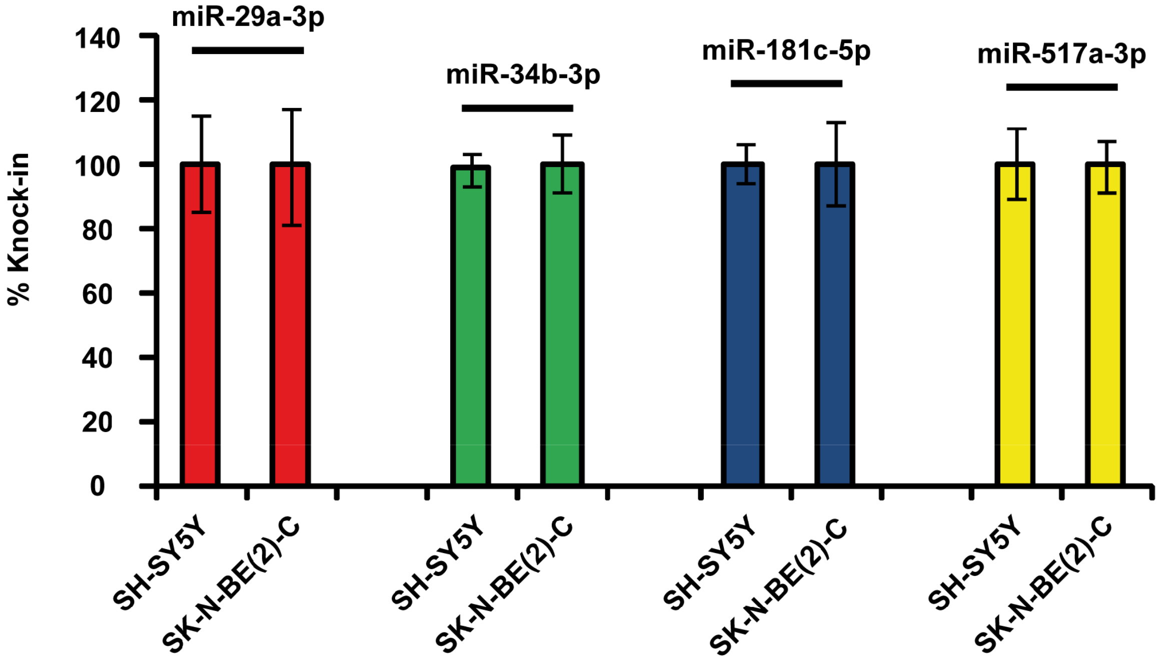

Supplementary Figure S3: MiR-29a-3p, miR-34b-3p, miR-181c-5p, miR-517a-3p knock-in efficiencies (%) after transfection (time point 24h) of SK-N-BE(2)-C and SH-SY5Y with specific miRNA mimics. Data are reported as mean ± standard deviation of three independent experiments.

DNMT3A vs miR-29a-3p

```
     3' auuGGCUAAAGU-CUACCACGAu 5' hsa-miR-29a
           |||| ||||  ||||||||
 847:5' aacCCGACUUCAUAAUGGUGCUu 3' DNMT3A
```

DNMT3B vs miR-29a-3p

```
     3' auuggcuaaagucuaCCACGAu 5' hsa-miR-29a
                       ||||||
 217:5' ggagcagccuaacacGGUGCUc 3' DNMT3B
```

```
     3' auuggcuaaagucuaCCACGAu 5' hsa-miR-29a
                       ||||||
1054:5' aaaacugcaaagcucGGUGCUc 3' DNMT3B
```

```
     3' auUGGCUAAAGUCUACCACGAu 5' hsa-miR-29a
          ||:  | |:|  |||||||
1189:5' uuACUCUUCUUA-CUGGUGCUa 3' DNMT3B
```

CDK6 vs miR-29a-3p

```
     3' auuggcuaAAGUCUACCACGAu 5' hsa-miR-29a
                | || ||||||||
8693:5' cuggcuugUCCACAUGGUGCUc 3' CDK6
```

```
     3' auUGGCUAA--AGUC--UACCACGAu 5' hsa-miR-29a
          || || |  || |   |||||||
9120:5' cuACAGAAUGCUCUGUUUUGGUGCUu 3' CDK6
```

```
     3' auuggcuaaagucuaCCACGAu 5' hsa-miR-29a
                       ||||||
9874:5' cuggaugucuuacugGGUGCUa 3' CDK6
```

E2F3 vs miR-34b-3p

```
     3' uaccgucaCCUCAAUCACUAAc 5' hsa-miR-34b
                ||:||  ||||||
3002:5' uuuccuacGGGGUACGUGAUUu 3' E2F3
```

BCL2 vs miR-34b-3p

```
     3' uaccgucaccUCAAUCACUAAc 5' hsa-miR-34b
                  ||  |||||||
3484:5' gggaaaaauuAGGAAGUGAUUa 3' BCL2
```

BCL2 vs miR-181c-5p

```
     3' ugaguggcuguccaaCUUACAa 5' hsa-miR-181c
                       ||||||
1063:5' ggcagugugguucccGAAUGUc 3' BCL2
```

```
     3' ugaguggcuguccaaCUUACAa 5' hsa-miR-181c
                       ||||||
1737:5' ucuguauuaacuuugGAAUGUa 3' BCL2
```

Supplementary Figure S4: Candidate miRNAs' binding sites retrieved by miRanda-mirSVR (http://www.microrna.org/microrna/home.do).

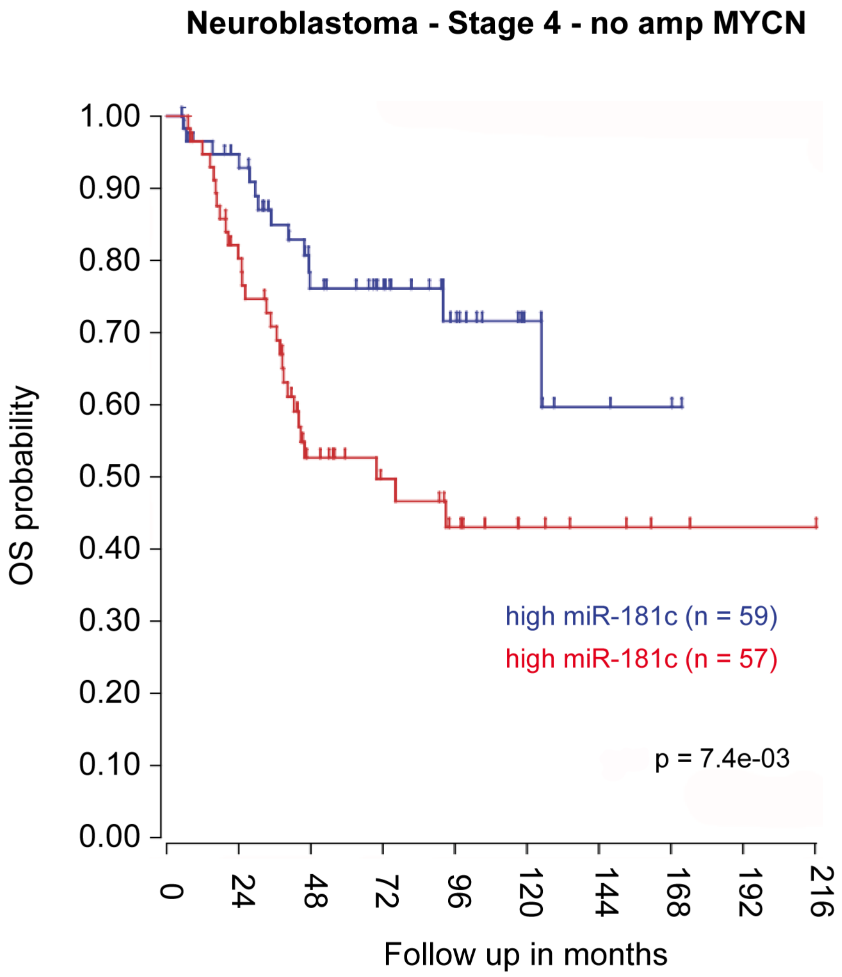

Supplementary Figure S5: miR-181c expression and stage 4 neuroblastoma patients' overall survival (OS). Stage 4 neuroblastoma patients with no amplified MYCN and lower miR-181c's expression have a worse prognosis with respect to the same patients with higher miR-181c expression. The cut off modus for miR-181c expression to draw Kaplan-Meier curves derives from the scan setting.

Supplementary Table S1: DE miRNAs from TLDA data. Not DE: not differentially expressed

See Supplementary File 1

Supplementary Table S2: Selection of DE miRNAs, based on their potential involvement in neuroblastoma (DE: differentially expressed; TS: tumor suppressor)

See Supplementary File 2

Supplementary Table S3: Detailed data on the methylation status within the CpG islands analyzed. For each genomic region investigated, percentage of methylation (%) of single CpG and the mean value are indicated for each cell line, both in controls (C) and after 5'-AZA treatment (Aza)

See Supplementary File 3

Supplementary Table S4: Selection of candidate miRNA targets

See Supplementary File 4

Supplementary Table S5: Target miRNA's binding site conservation (by TargetScan release 7.1)

miRNA	miRNA target	Conserved miRNA binding site (within target's 3'-UTR)	seed match
hsa-miR-29a-3p	**DNMT3A**	862-868	7mer-m8
hsa-miR-29a-3p	**DNMT3A**	1305-1311	7mer-m8
hsa-miR-29a-3p	**DNMT3A**	5559-5565	7mer-1A
hsa-miR-29a-3p	**DNMT3B**	1202-1209	8mer
hsa-miR-29a-3p	**CDK6**	9138-9144	7mer-m8
hsa-miR-29a-3p	**CDK6**	9889-9895	7mer-1A
hsa-miR-181c-5p	**BCL2**	1752-1758	7mer-1A
hsa-miR-181c-5p	**BCL2**	2896-2903	8mer

Supplementary Table S6: *Hub nodes* within candidate miRNA targets' network

Gene symbol	Betweenness	Closeness	Degree	Stress
CCNE1	1806.717681	0.004739336	45	10734
TAF1	1413.695084	0.004950495	45	9058
E2F4	1245.067842	0.005235602	49	9002
E2F1	1215.444905	0.005128205	48	8058
E2F3	1156.78274	0.004694836	38	7036
CDK2	807.1252773	0.004830918	40	6286
CCND1	745.7136432	0.004784689	34	5194
MYC	692.4908549	0.004694836	33	5610
RB1	651.1623185	0.005	39	6384
HDAC1	627.2007473	0.004237288	22	5460
CDK6	595.552135	0.004273504	29	4264
CDKN1A	587.1190398	0.004878049	36	4952
RBL2	575.4836829	0.004878049	34	5362
CDK4	500.0384738	0.004405286	28	4146
HNF4A	433.1309052	0.004504505	30	3326
AR	390.4186935	0.004329004	23	2692
DNMT3A	333.0231604	0.003546099	17	3128
DNMT3B	296.9462477	0.003333333	20	2790
CDKN1B	231.6297088	0.004651163	30	2682
CDC2	203.2643065	0.004444444	24	2542
CBX5	186.4977572	0.004115226	15	2320
**Threshold**	147.95	0.0038	12.1	1213.27
