# Supplementary material for: Altered expression of miRNAs and methylation of their promoters are correlated in neuroblastoma
Source: Oncotarget. 2016 Nov 4;7(50):83330–41. doi: 10.18632/oncotarget.13090 (PMC5347773; doi:10.18632/oncotarget.13090)
Supplement: Supplementary file 2 [file oncotarget-07-83330-s002.docx]

| **Supplementary Table 1.** DE miRNAs from TLDA data. Not DE: not differentially expressed. | | | | | |
| --- | --- | --- | --- | --- | --- |
| **microRNA** | **Average RQ ACN** | **Average RQ GIMEN** | **Average RQ SH-SY5Y** | **Average RQ SK-N-SH** | **Average RQ SK-N-BE(2)-C** |
| hsa-let-7b | 0.58 | 0.48 | 1.66 | 0.98 | 0.93 |
| hsa-let-7g* | 1.48 | 3.18 | 0.98 | Not DE | 0.74 |
| hsa-let-7i* | 0.77 | 13.35 | Not DE | Not DE | 2.82 |
| hsa-miR-10a | 1.21 | 0.48 | 0.80 | 1.97 | Not DE |
| hsa-miR-10b | 1.17 | 1.94 | 0.81 | 1.97 | Not DE |
| hsa-miR-15a | 0.27 | 2.06 | 0.38 | 1.03 | Not DE |
| hsa-miR-15b | 0.59 | 0.24 | 1.61 | 0.97 | Not DE |
| hsa-miR-17* | 11.82 | 6.70 | 0.93 | 0.55 | 1.40 |
| hsa-miR-21 | 1.15 | 0.98 | 0.40 | 1.00 | 1.85 |
| hsa-miR-21* | 1.47 | 3.37 | 0.47 | 0.55 | 1.43 |
| hsa-miR-22 | 2.36 | 3.91 | 0.37 | 1.00 | 3.46 |
| hsa-miR-29a | 1.22 | 1.92 | 0.84 | 1.93 | 1.88 |
| hsa-miR-29b | 0.60 | 1.88 | 0.42 | 4.03 | 0.93 |
| hsa-miR-29c | 0.59 | 1.92 | 1.64 | Not DE | 0.93 |
| hsa-miR-30b | 1.22 | 0.48 | 0.80 | 0.96 | 1.86 |
| hsa-miR-34a | 2.41 | 1.98 | Not DE | 2.01 | Not DE |
| hsa-miR-34b | Not DE | 7.21 | 0.06 | Not DE | 0.70 |
| hsa-miR-34c-5p | 4.65 | 1.90 | 0.20 | 0.49 | Not DE |
| hsa-miR-92a-1* | 2.98 | 0.83 | 0.96 | 0.54 | 0.71 |
| hsa-miR-99a* | 1.43 | 1.70 | 1.91 | 1.11 | 0.72 |
| hsa-miR-99b* | 0.37 | 0.84 | 0.96 | 0.55 | 0.70 |
| hsa-miR-100* | Not DE | 1.69 | 0.24 | Not DE | 0.72 |
| hsa-miR-101 | 0.58 | 0.94 | 0.80 | 1.99 | 1.87 |
| hsa-miR-125a-3p | 0.59 | 4.00 | 0.83 | 0.99 | 0.47 |
| hsa-miR-126 | 9.40 | 1.92 | 0.84 | 1.98 | 0.24 |
| hsa-miR-1274B | 1.46 | 0.42 | 0.48 | 1.10 | 1.45 |
| hsa-miR-1276 | 0.73 | 3.30 | 1.91 | 1.07 | 0.70 |
| hsa-miR-1285 | 1.47 | 3.24 | 0.48 | 1.16 | 1.39 |
| hsa-miR-1290 | 1.48 | 27.69 | 0.24 | 0.55 | 1.45 |
| hsa-miR-1300 | 0.72 | 6.94 | 0.99 | 1.99 | 1.42 |
| hsa-miR-132 | 1.18 | 0.49 | 0.80 | 1.98 | 0.92 |
| hsa-miR-133a | Not DE | 1.94 | 0.84 | 0.48 | 0.23 |
| hsa-miR-135a | 0.29 | 0.48 | 0.79 | 1.00 | Not DE |
| hsa-miR-135b* | 0.37 | 1.68 | Not DE | Not DE | 0.72 |
| hsa-miR-136* | Not DE | 0.84 | 0.03 | 0.27 | 0.70 |
| hsa-miR-140-3p | 2.33 | 1.98 | 0.81 | 2.01 | 0.92 |
| hsa-miR-141 | 2.35 | 1.95 | 0.10 | 4.10 | 1.90 |
| hsa-miR-143 | 1.17 | 0.95 | 0.81 | 0.49 | 0.23 |
| hsa-miR-144* | 5.91 | 0.84 | 1.95 | Not DE | 0.36 |
| hsa-miR-146a | Not DE | 1.98 | 0.40 | 1.96 | 0.46 |
| hsa-miR-148b | 1.16 | 0.23 | 0.82 | 0.97 | Not DE |
| hsa-miR-150 | 0.58 | 17.48 | 0.20 | 1.95 | 0.94 |
| hsa-miR-152 | 0.58 | 1.95 | 0.40 | 0.98 | 1.87 |
| hsa-miR-154* | Not DE | Not DE | 0.46 | 0.14 | 0.35 |
| hsa-miR-155 | 0.59 | 0.98 | 0.05 | 3.93 | 1.82 |
| hsa-miR-181a-2* | 2.95 | 0.83 | 0.97 | 1.10 | 0.71 |
| hsa-miR-181c | 2.44 | 1.88 | 0.82 | 1.90 | 1.74 |
| hsa-miR-183* | Not DE | 0.81 | 3.75 | Not DE | 0.36 |
| hsa-miR-185 | 0.59 | 3.99 | 0.81 | 0.98 | 0.91 |
| hsa-miR-186 | 0.58 | 0.96 | 0.41 | 1.95 | Not DE |
| hsa-miR-190 | 1.19 | 1.94 | 1.64 | 0.98 | 1.88 |
| hsa-miR-198 | Not DE | 7.79 | Not DE | Not DE | Not DE |
| hsa-miR-202 | 4.78 | 1.88 | 3.33 | 7.35 | Not DE |
| hsa-miR-203 | Not DE | Not DE | 0.82 | 1.95 | 0.91 |
| hsa-miR-205 | Not DE | 1.93 | Not DE | Not DE | Not DE |
| hsa-miR-206 | Not DE | 0.83 | 3.84 | 0.27 | 1.43 |
| hsa-miR-212 | 0.59 | 0.96 | 0.81 | 0.98 | 3.71 |
| hsa-miR-213 | 1.48 | 3.34 | 1.95 | 1.08 | 0.70 |
| hsa-miR-222 | Not DE | 0.98 | 0.40 | 0.98 | 3.66 |
| hsa-miR-222* | 0.74 | 0.43 | 0.24 | Not DE | 1.45 |
| hsa-miR-28-3p | 2.39 | 1.95 | 0.41 | 0.98 | 0.92 |
| hsa-miR-296-3p | 0.58 | 3.99 | 0.42 | 3.80 | 0.97 |
| hsa-miR-299-5p | Not DE | 0.06 | 0.83 | 0.49 | 0.46 |
| hsa-miR-302b | 1.20 | 0.97 | 3.34 | 0.25 | 0.93 |
| hsa-miR-302c | 4.93 | 0.96 | 0.86 | 0.49 | 0.49 |
| hsa-miR-330-5p | 0.29 | Not DE | 1.68 | 3.96 | Not DE |
| hsa-miR-335* | 2.99 | 0.42 | 0.48 | 1.05 | 0.73 |
| hsa-miR-345 | 0.29 | 0.97 | 1.62 | 1.00 | 0.91 |
| hsa-miR-367 | Not DE | 0.46 | 1.65 | 1.98 | 15.12 |
| hsa-miR-370 | Not DE | 0.48 | 0.82 | 0.49 | 0.47 |
| hsa-miR-372 | Not DE | Not DE | 0.05 | Not DE | Not DE |
| hsa-miR-374b* | 2.93 | 0.20 | 0.96 | 0.55 | 1.45 |
| hsa-miR-376a | 4.78 | 0.94 | 0.84 | 0.48 | Not DE |
| hsa-miR-380-5p | Not DE | Not DE | 0.24 | 0.27 | 0.73 |
| hsa-miR-411* | Not DE | 0.75 | 0.47 | 0.27 | 0.35 |
| hsa-miR-424 | 1.21 | 1.98 | 0.81 | 0.99 | 1.91 |
| hsa-miR-431 | Not DE | 1.01 | Not DE | 0.48 | 0.23 |
| hsa-miR-449a | 2.40 | Not DE | 1.68 | 1.00 | Not DE |
| hsa-miR-450b-5p | 0.59 | 0.24 | 0.82 | 0.49 | 0.92 |
| hsa-miR-455-5p | 2.28 | 1.91 | 0.83 | 1.97 | 0.95 |
| hsa-miR-483-3p | Not DE | 0.42 | 3.84 | 0.28 | 0.71 |
| hsa-miR-483-5p | 1.19 | 2.01 | 6.65 | 0.99 | 0.23 |
| hsa-miR-485-3p | Not DE | 0.49 | 0.82 | 0.48 | 0.23 |
| hsa-miR-489 | 1.18 | 0.47 | 0.41 | 0.97 | 0.47 |
| hsa-miR-490-3p | Not DE | Not DE | 0.40 | 1.00 | 7.07 |
| hsa-miR-491-5p | 1.18 | 1.95 | 0.41 | 0.98 | 0.92 |
| hsa-miR-503 | 4.89 | 1.94 | 0.39 | 0.25 | Not DE |
| hsa-miR-508-3p | 2.38 | 0.98 | Not DE | 1.93 | 3.66 |
| hsa-miR-511 | 2.36 | Not DE | Not DE | 0.99 | Not DE |
| hsa-miR-512-3p | Not DE | 7.83 | 0.88 | 4.13 | Not DE |
| hsa-miR-517a | Not DE | 31.75 | 30.29 | 3.45 | 62.25 |
| hsa-miR-517c | 0.56 | 15.27 | 0.43 | Not DE | 30.32 |
| hsa-miR-520c-3p | 0.73 | 3.36 | 0.94 | 4.48 | 1.44 |
| hsa-miR-520D-3p | Not DE | 3.44 | 0.49 | 0.56 | 0.73 |
| hsa-miR-523 | Not DE | 3.72 | Not DE | 1.95 | Not DE |
| hsa-miR-542-5p | 2.43 | 3.98 | 1.63 | 0.97 | Not DE |
| hsa-miR-543 | Not DE | 0.21 | 0.97 | 0.27 | 0.73 |
| hsa-miR-545* | 0.73 | 0.42 | 0.97 | 1.09 | 0.70 |
| hsa-miR-548a-3p | 4.89 | Not DE | 1.61 | 8.00 | Not DE |
| hsa-miR-548d-3p | 1.17 | 0.90 | 0.20 | 0.98 | 3.75 |
| hsa-miR-550 | Not DE | 0.83 | Not DE | 4.26 | 1.45 |
| hsa-miR-574-3p | 1.19 | 0.25 | 0.40 | 0.97 | 0.46 |
| hsa-miR-576-3p | 1.20 | 1.87 | 0.80 | 1.93 | 1.86 |
| hsa-miR-584 | 2.94 | Not DE | 1.00 | 1.00 | 1.00 |
| hsa-miR-589 | 0.18 | 0.43 | 0.47 | 1.13 | 1.48 |
| hsa-miR-616 | 2.95 | 0.83 | 0.23 | 1.12 | 2.86 |
| hsa-miR-622 | Not DE | 6.92 | Not DE | Not DE | Not DE |
| hsa-miR-624 | 0.37 | 3.33 | Not DE | 1.10 | 1.45 |
| hsa-miR-627 | 0.58 | 7.93 | 0.84 | Not DE | 1.93 |
| hsa-miR-628-5p | 2.38 | 0.96 | 0.81 | 1.95 | 0.93 |
| hsa-miR-645 | 0.18 | Not DE | 0.96 | 1.11 | 1.49 |
| hsa-miR-671-3p | 0.30 | 0.49 | 0.82 | 1.98 | 0.92 |
| hsa-miR-720 | 0.73 | 0.43 | 0.23 | 1.09 | 0.70 |
| hsa-miR-766 | 0.36 | 1.68 | 0.96 | 1.12 | 1.44 |
| hsa-miR-769-5p | 0.36 | 0.85 | 1.94 | 0.54 | 1.45 |
| hsa-miR-873 | 1.19 | 3.86 | 0.82 | 0.98 | 0.90 |
| hsa-miR-886-3p | 4.72 | Not DE | Not DE | Not DE | 14.72 |
| hsa-miR-886-5p | 1.18 | Not DE | 0.01 | Not DE | 3.63 |
| hsa-miR-922 | Not DE | 13.59 | 0.12 | Not DE | Not DE |
| hsa-miR-939 | 1.46 | 30.37 | 0.49 | Not DE | Not DE |
| hsa-miR-1183 | Not DE | 3.92 | 0.47 | Not DE | 0.66 |
| hsa-miR-1197 | Not DE | 0.84 | 0.48 | 0.27 | 0.35 |
| hsa-miR-1244 | 0.73 | 26.69 | 0.49 | 1.09 | 1.44 |
| hsa-miR-1248 | 1.49 | 3.42 | 0.97 | 1.12 | 0.73 |
| hsa-miR-1254 | 0.36 | 3.41 | 0.97 | 0.55 | 1.42 |
| hsa-miR-1255B | 1.47 | 6.44 | 0.49 | 1.07 | 1.51 |
