# Supplementary material for: Altered expression of miRNAs and methylation of their promoters are correlated in neuroblastoma
Source: Oncotarget. 2016 Nov 4;7(50):83330–41. doi: 10.18632/oncotarget.13090 (PMC5347773; doi:10.18632/oncotarget.13090)
Supplement: Supplementary file 3 [file oncotarget-07-83330-s003.docx]

**Supplementary Table 2.** Selection of DE miRNAs, based on their potential involvement in neuroblastoma (DE: differentially expressed; TS: tumor suppressor).

| **#** | **microRNA** | **Average RQ ACN** | **Average RQ GIMEN** | **Average RQ SH-SY5Y** | **Average RQ SK-N-SH** | **Average RQ SK-N-BE(2)-C** | **Validated CpG islands (literature: PMID)** | **Number of Predicted CpG islands** | **Neuroblastoma expression trend (literature)** | **miR2Disease cancer involvement** | **Potential role in cancer (literature)** | **MYCN functional relation based on literature** |
| --- | --- | --- | --- | --- | --- | --- | --- | --- | --- | --- | --- | --- |
| 1 | hsa-miR-22 | 2.36 | 3.91 | 0.37 | 1.00 | 3.46 | Not methylated validated CpG island (PMID: 19807731) | 2 | N/A | Upregulated in breast cancer (PMID: 19414598) and in AML (PMID: 18056805). Downregulated in lung cancer (PMID: 19654003) | N/A | N/A |
| 2 | hsa-miR-29a | 1.22 | 1.92 | 0.84 | 1.93 | 1.88 | N/A | N/A | Downregulated (PMID: 19584290) | Downregulated in neuroblastoma (PMID: 19584290) | TS | MYCN is a validated target (PMID: 21654684) |
| 3 | hsa-miR-34a | 2.41 | 1.98 | Not DE | 2.01 | Not DE | Validated and Methylated in renal cell cancer and sarcoma (PMIDs: 23321515, 25773680) | 2 | N/A | Downregulated in colon and pancreatic cancers (PMIDs: 19714243, 18519671) | TS | There is a negative correlation to MYCN mRNA levels (PMCID: 4467143) |
| 4 | hsa-miR-126 | 9.40 | 1.92 | 0.84 | 1.98 | 0.24 | Validated and hypermethylated in lung cancer. Activated by 5'-AZA treatment (PMIDs: 21702040, 19116145). | 2 | N/A | Downregulated in breast cancer and CRC (PMIDs: 18663744, 18834857) | TS | N/A |
| 5 | hsa-miR-140-3p | 2.33 | 1.98 | 0.81 | 2.01 | 0.92 | Upregulated in Gastric cancer after 5'-AZA treatment (PMID: 19923923). It has been shown to target HDAC4 (and probably other epigenetic players, such as DNMTs) (PMID: 17413852) | N/A | N/A | Downregulated in multiple mieloma (PMID: 19846888) | TS | N/A |
| 6 | hsa-miR-141 | 2.35 | 1.95 | 0.10 | 4.10 | 1.90 | Validated and reactivated by 5'-AZA treatment, miR-200c/miR-141. (PMIDs: 20682048, 20084174) | 1 | Not expressed | Downregulated in breast, kidney and lung cancers (PMIDs: 19759262, 18376396, 18925646 ) | TS | Not reported |
| 7 | hsa-miR-181c | 2.44 | 1.88 | 0.82 | 1.90 | 1.74 | N/A | N/A | Differentially expressed between MYCN-amplified and not-amplified tumors (PMID: 22936790) | Upregulated in AML. Downregulated in glioblastoma and lung cancer (PMIDs:16530703, 18478077, 16039986) | TS | Oncogenic miRNAs of miR-181 family are upregulated in neuroblastoma after MYCN silencing (PMID: 20466808) |
| 8 | hsa-miR-202 | 4.78 | 1.88 | 3.33 | 7.35 | Not DE | N/A | 1 | If overexpressed in neuroblastoma cell lines it suppresses endogenous N-myc protein, inhibits proliferation and clonogenic growth (PMID: 21654684). | Upregulated in breast cancer (PMID: 16103053). | TS | MYCN is a validated target (PMID: 21654684) |
| 9 | hsa-miR-455-5p | 2.28 | 1.91 | 0.83 | 1.97 | 0.95 | N/A | N/A | N/A | N/A | N/A | 2 |
| 10 | hsa-miR-508-3p | 2.38 | 0.98 | Not DE | 1.93 | 3.66 | N/A | N/A | N/A | N/A | TS | N/A |
| 11 | hsa-miR-517a | Not DE | 31.75 | 30.29 | 3.45 | 62.25 | Upregulated in gastric cancer after 5'-AZA treatment (PMID: 19923923). | N/A | N/A | N/A | TS | N/A |
| 12 | hsa-miR-576-3p | 1.20 | 1.87 | 0.80 | 1.93 | 1.86 | N/A | N/A | N/A | N/A | N/A | N/A |
