# Supplementary material for: Altered expression of miRNAs and methylation of their promoters are correlated in neuroblastoma
Source: Oncotarget. 2016 Nov 4;7(50):83330–41. doi: 10.18632/oncotarget.13090 (PMC5347773; doi:10.18632/oncotarget.13090)
Supplement: Supplementary file 4 [file oncotarget-07-83330-s004.docx]

**Supplementary Table 3.** Detailed data on the methylation status within the CpG islands analyzed. For each genomic region investigated, percentage of methylation (%) of single CpG and the mean value are indicated for each cell line, both in controls (C) and after 5’-AZA treatment (Aza).

| **Mir-29a** | **Genomic position (GRCh38/hg38)** | **Mean (%)** | **Treatment** | **CpG1 (%)** | **CpG2 (%)** | **CpG3 (%)** | **CpG4 (%)** |  |  |
| --- | --- | --- | --- | --- | --- | --- | --- | --- | --- |
| **ACN** | Chr7:130884862-130884892 | 85 | C | 86 | 94 | 100 | 59 |  |  |
|  |  | 80 | Aza | 80 | 89 | 97 | 56 |  |  |
| **GIMEN** |  | 90 | C | 100 | 100 | 100 | 61 |  |  |
|  |  | 73 | Aza | 75 | 80 | 87 | 50 |  |  |
| **SK-N-BE(2)-C** |  | 89 | C | 98 | 99 | 100 | 61 |  |  |
|  |  | 73 | Aza | 73 | 81 | 87 | 49 |  |  |
| **SK-N-SH** |  | 85 | C | 86 | 94 | 100 | 59 |  |  |
|  |  | 60 | Aza | 57 | 68 | 76 | 38 |  |  |
| **SH-SY5Y** |  | 84 | C | 87 | 93 | 96 | 61 |  |  |
|  |  | 58 | Aza | 56 | 66 | 70 | 44 |  |  |
|  |  |  |  |  |  |  |  |  |  |
| **Mir-34b/c** | **Genomic position (GRCh38/hg38)** | **Mean (%)** | **Treatment** | **CpG1 (%)** | **CpG2 (%)** | **CpG3 (%)** | **CpG4 (%)** |  |  |
| **ACN** | Chr11:111512560 -111512595 | 44 | C | 66 | 36 | 29 | 44 |  |  |
|  |  | 38 | Aza | 58 | 31 | 26 | 38 |  |  |
| **GIMEN** |  | 88 | C | 73 | 100 | 91 | 90 |  |  |
|  |  | 79 | Aza | 64 | 94 | 77 | 81 |  |  |
| **SK-N-BE(2)-C** |  | 93 | C | 86 | 100 | 94 | 94 |  |  |
|  |  | 76 | Aza | 71 | 85 | 71 | 77 |  |  |
| **SK-N-SH** |  | 56 | C | 88 | 32 | 31 | 74 |  |  |
|  |  | 44 | Aza | 78 | 29 | 26 | 44 |  |  |
| **SH-SY5Y** |  | 13 | C | 22 | 9 | 7 | 14 |  |  |
|  |  | 10 | Aza | 12 | 9 | 7 | 12 |  |  |
|  |  |  |  |  |  |  |  |  |  |
| **Mir-126** | **Genomic position (GRCh38/hg38)** | **Mean (%)** | **Treatment** | **CpG1 (%)** | **CpG2 (%)** | **CpG3 (%)** | **CpG4 (%)** | **CpG5 (%)** | **CpG6 (%)** |
| **ACN** | Chr9:136670573-136670611 | 46 | C | 48 | 56 | 48 | 69 | 27 | 28 |
|  |  | 43 | Aza | 46 | 56 | 44 | 65 | 23 | 25 |
| **GIMEN** |  | 84 | C | 91 | 95 | 88 | 90 | 71 | 67 |
|  |  | 66 | Aza | 69 | 76 | 67 | 74 | 55 | 56 |
| **SK-N-BE(2)-C** |  | 48 | C | 52 | 58 | 63 | 57 | 32 | 24 |
|  |  | 39 | Aza | 43 | 48 | 46 | 50 | 27 | 19 |
| **SK-N-SH** |  | 34 | C | 49 | 28 | 39 | 52 | 18 | 16 |
|  |  | 25 | Aza | 34 | 23 | 28 | 32 | 16 | 16 |
| **SH-SY5Y** |  | 86 | C | 92 | 95 | 87 | 91 | 71 | 79 |
|  |  | 53 | Aza | 57 | 61 | 47 | 63 | 43 | 47 |
|  |  |  |  |  |  |  |  |  |  |
| **Mir-181** | **Genomic position (GRCh38/hg38)** | **Mean (%)** | **Treatment** | **CpG1 (%)** | **CpG2 (%)** | **CpG3 (%)** | **CpG4 (%)** | **CpG5 (%)** |  |
| **ACN** | Chr19:13873901-13873940 | 59 | C | 92 | 91 | 58 | 34 | 22 |  |
|  |  | 47 | Aza | 73 | 71 | 46 | 28 | 18 |  |
| **GIMEN** |  | 94 | C | 97 | 97 | 94 | 85 | 95 |  |
|  |  | 62 | Aza | 66 | 64 | 62 | 57 | 63 |  |
| **SK-N-BE(2)-C** |  | 88 | C | 94 | 96 | 93 | 81 | 77 |  |
|  |  | 60 | Aza | 65 | 67 | 60 | 54 | 52 |  |
| **SK-N-SH** |  | 87 | C | 92 | 93 | 86 | 77 | 90 |  |
|  |  | 52 | Aza | 58 | 60 | 50 | 43 | 47 |  |
| **SH-SY5Y** |  | 92 | C | 95 | 96 | 94 | 81 | 92 |  |
|  |  | 49 | Aza | 57 | 52 | 46 | 46 | 47 |  |
|  |  |  |  |  |  |  |  |  |  |
| **Mir-200c** | **Genomic position (GRCh38/hg38)** | **Mean (%)** | **Treatment** | **CpG1 (%)** | **CpG2 (%)** | **CpG3 (%)** | **CpG4 (%)** | **CpG5 (%)** |  |
| **ACN** | Chr12:6963417-6963456 | 84 | C | 95 | 99 | 67 | 80 | 78 |  |
|  |  | 70 | Aza | 83 | 80 | 54 | 67 | 64 |  |
| **GIMEN** |  | 86 | C | 98 | 100 | 75 | 79 | 76 |  |
|  |  | 65 | Aza | 72 | 81 | 48 | 63 | 59 |  |
| **SK-N-BE(2)-C** |  | 89 | C | 97 | 98 | 78 | 87 | 84 |  |
|  |  | 60 | Aza | 69 | 75 | 46 | 56 | 57 |  |
| **SK-N-SH** |  | 86 | C | 97 | 100 | 71 | 82 | 80 |  |
|  |  | 54 | Aza | 64 | 70 | 40 | 48 | 49 |  |
| **SH-SY5Y** |  | 96 | C | 98 | 100 | 86 | 98 | 100 |  |
|  |  | 54 | Aza | 57 | 63 | 42 | 55 | 54 |  |
|  |  |  |  |  |  |  |  |  |  |
| **Mir-202** | **Genomic position (GRCh38/hg38)** | **Mean (%)** | **Treatment** | **CpG1 (%)** | **CpG2 (%)** | **CpG3 (%)** | **CpG4 (%)** | **CpG5 (%)** |  |
| **ACN** | Chr10:133247892-133247938 | 34 | C | 20 | 10 | 52 | 41 | 49 |  |
|  |  | 35 | Aza | 20 | 14 | 48 | 43 | 49 |  |
| **GIMEN** |  | 88 | C | 85 | 94 | 66 | 100 | 94 |  |
|  |  | 66 | Aza | 48 | 63 | 42 | 100 | 78 |  |
| **SK-N-BE(2)-C** |  | 88 | C | 86 | 90 | 71 | 100 | 93 |  |
|  |  | 73 | Aza | 69 | 71 | 56 | 91 | 76 |  |
| **SK-N-SH** |  | 85 | C | 81 | 85 | 67 | 100 | 92 |  |
|  |  | 53 | Aza | 46 | 47 | 45 | 69 | 55 |  |
| **SH-SY5Y** |  | 74 | C | 69 | 75 | 67 | 85 | 76 |  |
|  |  | 60 | Aza | 57 | 56 | 56 | 72 | 57 |  |
|  |  |  |  |  |  |  |  |  |  |
| **Mir-517** | **Genomic position (GRCh38/hg38)** | **Mean (%)** | **Treatment** | **CpG1 (%)** | **CpG2 (%)** | **CpG3 (%)** | **CpG4 (%)** |  |  |
| **ACN** | Chr19:536480027-536480057 | 80 | C | 79 | 80 | 88 | 64 |  |  |
|  |  | 78 | Aza | 72 | 87 | 90 | 63 |  |  |
| **GIMEN** |  | 96 | C | 92 | 96 | 100 | 97 |  |  |
|  |  | 77 | Aza | 75 | 78 | 79 | 78 |  |  |
| **SK-N-BE(2)-C** |  | 90 | C | 81 | 95 | 100 | 85 |  |  |
|  |  | 80 | Aza | 73 | 84 | 87 | 76 |  |  |
| **SK-N-SH** |  | 88 | C | 80 | 92 | 97 | 81 |  |  |
|  |  | 66 | Aza | 62 | 69 | 75 | 60 |  |  |
| **SH-SY5Y** |  | 95 | C | 94 | 96 | 100 | 90 |  |  |
|  |  | 72 | Aza | 76 | 73 | 77 | 63 |  |  |
