# Supplementary material for: Altered expression of miRNAs and methylation of their promoters are correlated in neuroblastoma
Source: Oncotarget. 2016 Nov 4;7(50):83330–41. doi: 10.18632/oncotarget.13090 (PMC5347773; doi:10.18632/oncotarget.13090)
Supplement: Supplementary file 5 [file oncotarget-07-83330-s005.docx]

**Supplementary Table 4**. Selection of candidate miRNA targets.

| **microRNA** | **Target** | **miRNA-target interaction** | **Queried Database** | **Biological function** |
| --- | --- | --- | --- | --- |
| miR-181c-5p | GATA6 | Validated | miRecords / Tarbase | It promotes colon cancer cell invasion (PMID: 21076612). |
|  | BCL2 | Validated | miRecords / Tarbase | Anti-apoptosis oncogene. Targeted BCL2 inhibition effectively inhibits neuroblastoma tumor growth (PMIDs: 22366560, 23173842, 18955968). |
|  | SIRT1 | Validated | miRecords / Tarbase | It can act as both tumor suppressor or oncogenic factor depending on the context and the study conditions (PMID: 21941620). |
|  | KIT | Validated | miRecords / Tarbase | The abnormality of the SCF/c-kit signaling pathway is closely related to certain tumors. Oncogene targeted for cancer therapy (PMIDs: 23127174, 23678293). |
| miR-29a-3p | CDK6 | Validated | miRecords / Tarbase | Regulator of cell cycle, involved in different cancers, e.g.: breast, colon and lung (PMIDs: 23948297, 25425654). |
|  | RAN | Validated | miRecords / Tarbase | Its function is related to PI3K/Akt/mTORC1 and Ras/MEK/ERK pathways activation. Involved in epithelial ovarian cancer cell survival (PMIDs: 22090358, 20942967). |
|  | DNMT3A | Validated | miRecords / Tarbase | Associated with hematological cancers (PMID: 25693834). |
|  | DNMT3B | Validated | miRecords / Tarbase | Dnmt3b promotes tumorigenesis *in vivo* as well as its overexpression contributes to a hypermethylator phenotype in human breast cancer cell lines (PMIDs: 2081977, 18221536). |
| miR-34b-3p | BCL2 | Validated | miRecords / Tarbase | Anti-apoptotic oncogene. Targeted BCL2 inhibition effectively inhibits neuroblastoma tumor growth (PMIDs: 22366560, 23173842, 18955968). |
|  | MYB | Validated | miRecords / Tarbase | Involved in leukemia, and activated in colon and breast cancers (PMIDs: 18574464, 20659323). |
|  | CCNE2 | Validated | miRecords / Tarbase | CCN2 inhibits lung cancer metastasis. Inhibition of connective tissue growth factor (CTGF/CCN2) in gallbladder cancer cells leads to decreased growth *in vitro* (PMIDs: 23175185, 23593935). |
|  | CDK4 | Validated | miRecords / Tarbase | It is a therapeutic target, activated in different cancers. Overexpressed in lung cancer. (PMID: 25716100, PMC: 3094221). |
|  | E2F3 | Validated | miRecords / Tarbase | Associated with prostate cancer outcome, and overexpressed in bladder cancer (PMIDs: 15184867, 14716298). |
| miR-517a-3p | OLFM3 | Predicted | miRecords - Clipseq - Mirgator | OLFM3 down-regulation can increase the anoikis of cells in culture. Involved in cell-cell and cell-matrix adhesion (PMIDs: 22281030, 16115881). |
|  | WEE1 | Predicted | miRecords - Clipseq - Mirgator | Kinase target for cancer therapy, involved in the regulation of genome instability (PMIDs: 23727417, 24013427). |
|  | TNIP1 | Predicted | miRecords - Clipseq - Mirgator | NAF-1 is central to human breast cancer proliferation and growth (PMID: 23959881). |
|  | IFNAR1 | Predicted | miRecords - Clipseq - Mirgator | Promotes cell survival in cancer as well as colorectal cancer cells survival (PMIDs: 25546690, 23959176). |
